# Sex, Age, and Handedness Modulate the Neural Correlates of Active Learning

**DOI:** 10.3389/fnins.2019.00961

**Published:** 2019-09-11

**Authors:** Sangeeta Nair, Rodolphe E. Nenert, Jane B. Allendorfer, Adam M. Goodman, Jennifer Vannest, Daniel Mirman, Jerzy P. Szaflarski

**Affiliations:** ^1^Department of Neurology, The University of Alabama at Birmingham, Birmingham, AL, United States; ^2^Department of Psychology, The University of Alabama at Birmingham, Birmingham, AL, United States; ^3^Department of Pediatrics, Division of Neurology, Cincinnati Children’s Hospital Medical Center, Pediatric Neuroimaging Research Consortium, University of Cincinnati College of Medicine, Cincinnati, OH, United States; ^4^Department of Psychology, University of Edinburgh, Edinburgh, United Kingdom

**Keywords:** associate learning, fMRI, handedness, sex, age, verbal memory

## Abstract

**Background:**

Self-generation of material compared to passive learning results in mproved memory performance; this may be related to recruitment of a fronto-temporal encoding network. Using a verbal paired-associate learning fMRI task, we examined the effects of sex, age, and handedness on the neural correlates of self-generation.

**Methods:**

Data from 174 healthy English-speaking participants (78M, 56 atypically handed; ages 19–76) were preprocessed using AFNI and FSL. Independent component analysis was conducted using GIFT (Group ICA fMRI Toolbox). Forty-one independent components were temporally sorted by task time series. Retaining correlations (*r* > 0.25) resulted in three task-positive (“generate”) and three task-negative (“read”) components. Using participants’ back-projected components, we evaluated the effects of sex, handedness, and aging on activation lateralization and localization in task-relevant networks with two-sample *t*-tests. Further, we examined the linear relationship between sex and neuroimaging data with multiple regression, covarying for scanner, age, and handedness.

**Results:**

Task-positive components identified using ICA revealed a fronto-parietal network involved with self-generation, while task-negative components reflecting passive reading showed temporo-occipital involvement. Compared to older adults, younger adults exhibited greater task-positive involvement of the left inferior frontal gyrus and insula, whereas older adults exhibited reduced prefrontal lateralization. Greater involvement of the left angular gyrus in task-positive encoding networks among right-handed individuals suggests the reliance on left dominant semantic processing areas may be modulated by handedness. Sex effects on task-related encoding networks while controlling for age and handedness suggest increased right hemisphere recruitment among males compared to females, specifically in the paracentral lobe during self-generation and the suparmarginal gyrus during passive reading.

**Implications:**

Identified neuroimaging differences suggest that sex, age, and handedness are factors in the differential recruitment of encoding network regions for both passive and active learning.

## Introduction

Active and passive learning are the mainstays of acquiring new knowledge. Active learning involves thoughtful analysis of, and engagement with, new content. Reading complete information is a form of passive learning that does not require engaging with the material. The benefits of active learning have been well studied, and active engagement in the classroom has been widely shown to improve retention of information, academic achievement, and self-esteem (Schefft and Biederman, 1990; Springer et al., 1999; Prince, 2004).

### Active Learning

Actively learning and memorizing new verbal information has been shown to improve retention of information compared to passive reading (McDaniel et al., 1988; Olofsson and Nilsson, 1992). Self-generation is a type of active learning that involves the discovery and production of an item based on incomplete information. During self-generation, the individual generates the target item based on a cue, or a piece of information that aids in retrieval of the target item (Jacoby, 1978). For example, the use of mnemonic devices can aid in retrieval and bolster memory of target items among aging adults (Hill et al., 1990; Derwinger et al., 2005). There is a consensus that such active participation leads to improved outcomes compared to passive participation among healthy individuals across a range of domains including memory, coordination of selective attention, mood state, self-esteem, and generalization of new knowledge (Schefft and Biederman, 1990; Walsh et al., 1995; Markant et al., 2016). Self-generation techniques have been shown to also improve memory across numerous clinical groups, including Alzheimer’s disease and dementia (Lipinska et al., 1994; Souliez et al., 1996; Barrett et al., 2000), Parkinson’s disease (Barrett et al., 2000), traumatic brain-injury (Schefft et al., 2008a), epilepsy (Schefft et al., 2008b), and aphasia (Marshall et al., 1994).

The benefit of active over passive learning is due to the nature of the process itself: generation is a problem-solving task where one obtains the solution by engaging in a series of operations (e.g., finding relations among cues). Several cognitive mechanisms by which generation improves retention have been proposed (Otten et al., 2001; Craik, 2002; Nyberg, 2002). For example, the process of active self-generation increases distinctiveness or relevance of target words compared to passively read words and thus increases retention of the target words (McDaniel et al., 1988; Walsh et al., 1995). Or, enhanced memory may be the result of improved self-esteem by having successfully solved a problem (Olofsson and Nilsson, 1992). Enhanced retention and memory from self-generation may also be due to a deeper level of cognitive processing required for active compared to passive reading (Craik and Lockhart, 1972; Craik, 2002; Lespinet-Najib et al., 2004).

### Neural Correlates of Active Learning and Deep Semantic Processing

Neuroimaging studies have reported recruitment of a frontal and medial temporal encoding network during tasks requiring a deeper level of processing (Otten et al., 2001; Nyberg, 2002; Binder and Desai, 2011). Across a range of tasks that demand deep semantic processing and attention, greater activity in the left prefrontal cortex has been associated with better memory performance (Kapur et al., 1994; Shallice et al., 1994; Demb et al., 1995; Buckner et al., 1999; McDermott et al., 1999). Additional cortical and subcortical regions implicated in successful encoding of new information include bilateral lingual, fusiform, inferior frontal, and parahippocampal gyri, premotor and medial parietal cortices, anterior cingulate cortex, thalami, and left insula (Kapur et al., 1995; Fletcher and Henson, 2001; Szaflarski et al., 2004; Kim, 2011). One neuroimaging study examined brain areas involved in shallow vs. deep semantic processing to suggest the bilateral inferior prefrontal cortex and left anterior and posterior hippocampus to be differentially activated depending on the depth of processing, particularly in verbal memory encoding (Otten et al., 2001). Successful non-semantic encoding (using an alphabetical task) was shown to recruit a specific subset of these brain regions (Otten et al., 2001). Similar studies employing tasks isolating semantic vs. shallow processing have found that deeper semantic processing was associated with increased activity in left prefrontal regions (Kapur et al., 1994; Grady et al., 1998; Buckner et al., 2000; Cabeza and Nyberg, 2000). It has been suggested that encoding of verbal/semantic information may be left lateralized, while non-verbal encoding (e.g., scenes) may involve bilateral recruitment (Nyberg et al., 1996).

Lateralization effects have also been seen with episodic memory encoding and retrieval as described via the HERA model (Hemispheric Encoding/Retrieval Asymmetry; Tulving et al., 1994; Nyberg et al., 1996). Originally described in young participants, the HERA model describes hemispheric asymmetry of the prefrontal cortex (PFC): specifically, greater involvement of the left PFC during encoding of episodic long-term memory and recruitment of right PFC during retrieval of episodic long-term memory (Tulving et al., 1994). However, the HERA model did not generalize to older populations, where bilateral PFC involvement was seen during both encoding and retrieval stages of an episodic memory task (Cabeza et al., 1997a, b). Lateralization may also be related to age (Szaflarski et al., 2006, 2012; Allendorfer et al., 2012b), as described by the HAROLD (Hemispheric Asymmetry Reduction in OLder ADults) model of functional lateralization (Cabeza, 2002; Nenert et al., 2018; see section “Age-Related Changes in Self-Generation”).

Active learning is a top–down approach to problem solving that, depending on the task, can involve a range of cognitive functions including attention, cognitive effort, item distinctiveness, working memory, and semantic and conceptual processing (Rosner et al., 2013). It engages a wide range of cognitive functions, suggesting distributed and highly connected networks. Theories of executive control and working memory suggest prefrontal regulation of posterior brain activity (Miller and Cohen, 2001; Shimamura, 2008), and activation of a broad fronto-temporal network has been supported by neuroimaging findings (Otten et al., 2001; Nyberg, 2002; Kirchhoff and Buckner, 2006; Qin et al., 2007). Specifically, studies of encoding and retrieval have reported recruitment of inferior frontal gyrus (IFG; long-term memory; Poldrack et al., 1999; Baker et al., 2001; Bookheimer, 2002; Liakakis et al., 2011), dorsolateral prefrontal cortex (manipulation of visuospatial information and long-term memory formation; Frith et al., 1991; Paller and Wagner, 2002), cingulate gyrus (conflict monitoring, attention; van Veen et al., 2001; Botvinick et al., 2004), middle temporal gyrus (verbal or item analysis; Cabeza and Nyberg, 2000; Binder et al., 2009), and parahippocampal areas (memory; Otten et al., 2001). Further, a meta-analysis of successful memory effects indicated broad involvement of the fronto-temporal network including the left inferior frontal cortex/insula, bilateral fusiform cortex, and left medial temporal cortex (Kim, 2011).

Finally, one study examined active learning processes via paired-associates encoding and verbal self-generated responses in an fMRI task (Vannest et al., 2012), and the results were consistent with the depth of processing literature (Tulving et al., 1994). The nature of the self-generation condition forces participants to access knowledge of various semantic elements of the cue and target words, leading to deeper cognitive processing than the reading condition. These results supported previous behavioral findings of improved memory performance in self-generation, as well as participation of left lateralized fronto-parietal areas during active encoding (Vannest et al., 2012, 2015).

### Sex Differences of Memory and Language: Lateralization and Depth of Processing

Sex differences in the lateralization of memory and language domains have been previously identified (Shaywitz et al., 1995). One study investigating phonological processing found that males showed strong left lateralized activation in the IFG while females had more diffuse, bilateral involvement (Shaywitz et al., 1995). In semantic tasks, females typically show widespread right hemispheric involvement whereas males show strongly left lateralized activity (Kimura, 1983; Pugh et al., 1996; Jaeger et al., 1998; Phillips et al., 2000). While cognitive strategies may differ between sexes, males and females frequently perform similarly on behavioral measures (Shaywitz et al., 1995; Berenbaum et al., 1997; Weiss et al., 2006). However, females do show some advantages in verbal memory, verbal fluency and production, and tasks with meaningful, semantic content (Kimura and Clarke, 2002; Kimura and Seal, 2003; Andreano and Cahill, 2009). One study examined the effect of sex when controlling for memory performance in a verb generation task (Allendorfer et al., 2012a). Their results suggested that both sexes display similar activation patterns when controlling for in-scanner performance, though minor differences were observed (Allendorfer et al., 2012a).

An event-related potential (ERP study) examining the dynamics of passive language processing found that the temporal characteristics of the early stages of lexical-semantic encoding are similar among both sexes (Wirth et al., 2006). However, differences in higher-order, controlled semantic processing suggest females engage in a deeper level of processing compared to males, demonstrating faster processing of related words (as measured by the N400). There is also support for differential organization of information across sexes during verbal learning tasks (Kramer et al., 1988; Sunderaraman et al., 2013) suggesting different processing strategies during encoding (Mulligan and Lozito, 2004).

### Age-Related Changes in Self-Generation

Aging has been suggested to impact the lateralization of language networks underlying semantic processing (Szaflarski et al., 2006, 2012; Allendorfer et al., 2012b). The HAROLD model proposes that language functions in the brain become less lateralized with age, which may be due to a compensatory mechanism during aging (compensation view), or increased difficulty recruiting domain-specific neural networks (dedifferentiation view; Cabeza, 2002). Initially developed with respect to pre-frontal activity, the HAROLD model may also be generalized to temporal and parietal brain areas (Bellis et al., 2000; Grady et al., 2000, 2002).

An investigation into age-related changes in the neural bases of encoding found that although overall memory performance of self-generated words decreased among older individuals, self-generated words were better remembered compared to read words across all age groups (Vannest et al., 2015). In this study, age-related decreases in connectivity of networks associated with self-generation did not correspond to a decrease in memory performance, suggesting that these networks may be less affected by age-related dedifferentiation (Cabeza, 2002). One example of dedifferentiation may be reduced lateralization seen in older adults during implicit memory tests (Bergerbest et al., 2008), where they tend to show bilateral brain activation in domains that younger adults show strong unilateral activation (Logan et al., 2002; Rosen et al., 2002; Morcom et al., 2003; Szaflarski et al., 2006).

### Impact of Handedness on Semantic Encoding and Retrieval

Handedness and language lateralization are linked genetically (Szaflarski et al., 2002, 2012). One study theorized that atypical-handers (left- or mixed-handers) may have a retrieval advantage over right handers due to higher dependence of these processes on interhemispheric communication (Christman and Propper, 2001; Prichard et al., 2013). Increased right hemispheric engagement among atypical-handers is supported by studies showing a relationship between handedness and corpus callosum volume where larger volume is associated with atypical-handedness (Habib et al., 1991; Witelson and Goldsmith, 1991; Luders et al., 2010). While encoding of verbal information involves left prefrontal areas, retrieval of that information recruits right prefrontal areas (Tulving et al., 1994). This suggests that increased access to the right hemisphere may aid in retrieval, consistent with the HERA model (Tulving et al., 1994; Propper and Christman, 2004; Propper et al., 2005; Chu et al., 2012). However, a recent study did not find any handedness-related behavioral differences in working memory tasks though their findings of an advantage among atypical handers during episodic retrieval were consistent with previous literature (Sahu et al., 2016).

In view of the available studies, our main objective was to examine the neural correlates of semantic learning during self-generation and investigate the role sex may play in brain network engagement during this process. We investigate these questions within a two-level analysis framework. The first level of analysis identified task-related networks using a hypothesis-independent source separation technique, independent component analysis (ICA), and by temporally sorting components based on the task time series. In the second level analyses, we compared subject component maps for the identified task-related networks in a series of subsequent hypothesis-driven analyses regarding the role sex, age, and handedness may play in the recruitment of these task-related networks (Bartels and Zeki, 2005). We hypothesized that results would show sex differences in support of a left lateralized language network among males and more widespread, bilateral processing among females, associated with similar behavioral outcomes. The present study also investigated the role age and handedness may play in active encoding, and whether any differences remain among sexes when taking these variables into account. We hypothesized that the self-generation process would be affected by these factors in such a way that age would affect the robustness or degree of connectivity in fronto-parietal, task-related networks. In addition, potential compensatory mechanisms may play a role in performance if these factors impact self-generation and active encoding.

## Materials and Methods

### Participants

Participants were 174 native English-speaking adults (96 female; 56 atypically handed; ages 19–76) with no history of neurological or psychiatric disorders (Table 1). Handedness was determined using the derived laterality quotient from the Edinburgh Handedness Inventory (Oldfield, 1971). Participants were coded categorically as follows: atypically handed from −100 to +49 and right-handed from +50 to +100. The Institutional Review Boards at the University of Cincinnati, the Cincinnati Children’s Hospital Medical Center, and the University of Alabama at Birmingham approved this project (NIH R01-NS04828), and all participants provided written informed consent.

**TABLE 1:** Participant demographics.

	**Male participants (*N* = 78)**	**Female participants (*N* = 96)**	**All participants (*N* = 174)**
**Age**			
Mean (SD)	40.71 (14.1)	41.66 (15.0)	41.23 (14.6)
Min – max	19 – 74	19 – 76	19 – 76
**Handedness (#)**			
Right	54	64	118
Atypical	24	32	56
**In-scanner performance (%)**			
**Read**			
Correct	71.3%	87.9%	80.6%
Incorrect	24.0%	11.8%	17.1%
No response	4.8%	0.25%	2.3%
**Generate**			
Correct	58.2%	71.0%	65.3%
Incorrect	31.2%	20.0%	24.9%
No response	10.7%	9.0%	9.7%
**Post-test accuracy (%)**			
Read	70.7%	74.0%	72.5%
Generate	73.7%	76.5%	75.2%

### Materials

Related word pairs were chosen from previous studies, with all included words under 6 letters long (Basso et al., 1994; Schefft et al., 2008a, b; Siegel et al., 2012). The 60 selected word pairs were evenly distributed across 5 relationship classes: associates (e.g., *lock – key*), category members (e.g., *saucer – bowl*), synonyms (e.g., *street – road*), antonyms (e.g., *hot – cold*), and rhymes (e.g., *care – dare*) (Siegel et al., 2012).

### Paired-Associate Learning Task and Recognition

**FIGURE 1:**
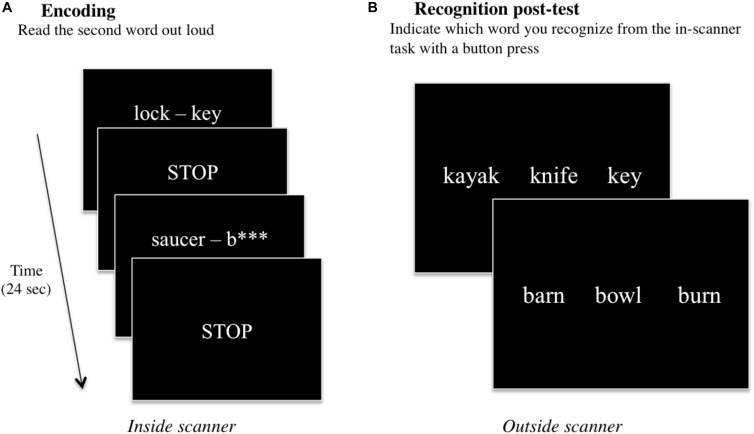
A schematic of the two parts of the verbal paired-associate learning task consisting of **(A)** an in-scanner encoding task, and a **(B)** post-fMRI recognition test. During the encoding task, word pairs are presented on the screen for 6 s, and participants are instructed to read (or generate) the second word of the word pair aloud. The STOP screen instructs participants to stop talking while three image volumes are acquired for a total of 6 s. This continues for all 60 word-pair stimuli, for a total of 12 min. After scanning, participants conduct a recognition post-test in a nearby facility testing room. Participants are presented with three words: one target word they had been exposed to during the in-scanner encoding task, and two foils, and participants are instructed to indicate which of the three words they recognize from the task.

This fMRI task was previously utilized by our group (Basso et al., 1994; Schefft et al., 2008b; Siegel et al., 2012; Vannest et al., 2012, 2015). Also data from some of the participants were included in previous studies (Siegel et al., 2012; Vannest et al., 2012, 2015). Briefly, the verbal paired-associate learning task was presented during the fMRI scanning session, and a recognition post-test was administered in a testing room thereafter (see section “fMRI Data Preprocessing”). During the in-scanner task, 60 word pairs were presented either in full (e.g., *spider – web*) or with the second word partially missing (e.g., *bed – p^∗∗∗∗∗^*), and participants were instructed to say the second word aloud (Figure 1A). In the “read” condition (e.g., *spider – web*), participants simply read the second word in the pair aloud. In the “generate” condition (e.g., *bed – p^∗∗∗∗∗^*), participants had to first self-generate the target word and then say it aloud. In-scanner responses were monitored and transcribed.

After the scanning session, participants performed a recognition test evaluating their memory of the second word in each word pair that was presented during the fMRI task. All 60 words presented during the fMRI task across both “read” and “generate” conditions (30 words per condition) were included in the post-test in a three-item forced-choice format. The target word and two foils were presented on a computer screen, and participants indicated which of the three words they recognized from the in-scanner task with a key press on the computer (Figure 1B). The post-test was self-paced; the test would advance to the next set of three items once the subject had responded. Post-test performance scores were analyzed for any statistical differences using Wilcoxon signed-rank tests for “read” vs. “generated” words across all participants, and among males vs. females, atypically handed vs. right handed participants, and older adults vs. younger (see section “Performance Data”).

### MRI Acquisition

**FIGURE 2:**
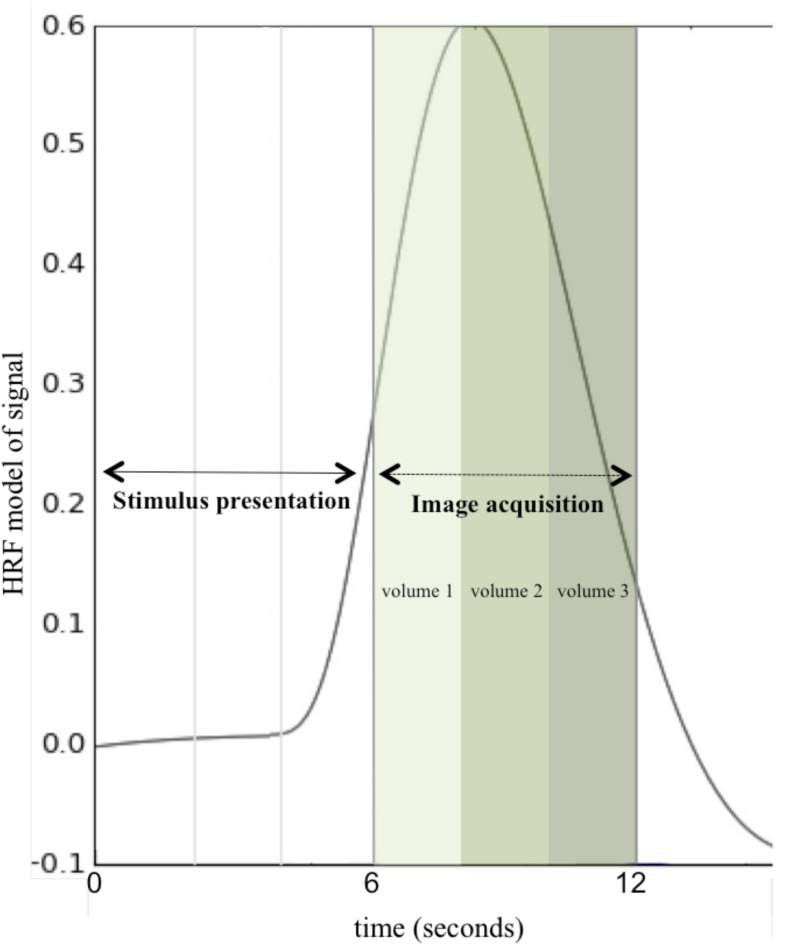
Hemodynamics Unrelated to Sounds of Hardware (HUSH) Image Acquisition. A schematic of one stimulus presentation and image acquisition period (total of 12 s) using a clustered-sparse temporal acquisition, HUSH. Stimulus is presented for the first 6 s, followed by acquisition of three image volumes with a TR of 2 s. This is repeated for all 60 word-pair stimuli for 12 min and a total of 180 image volumes (HRF, Hemodynamic response function).

Anatomical and functional MRI data were acquired for the 174 participants included in analysis across two scanners: a 3T Philips Achieva MRI scanner at the Cincinnati Children’s Hospital in the Imaging Research Center (151 participants) and a 3T head-only Siemens Magnetom Allegra MRI scanner at the University of Alabama at Birmingham provided by the Civitan Functional Neuroimaging Laboratory (23 participants). Across both scanners, data were acquired using a clustered-sparse temporal image acquisition called Hemodynamics Unrelated to Sounds from Hardware (HUSH; Schmithorst and Holland, 2004; see Figure 2). In addition to allowing for the ability to record overt spoken responses inside the scanner, the HUSH partially silent event-related design takes advantage of the delayed response of the hemodynamic response function (HRF). The positive peak of the HRF occurs approximately 4 to 6 s post-stimulus presentation and response (Buxton et al., 2004), allowing us to capture activity taking place seconds preceding data collection. Scanner type was used as a covariate in all analyses.

#### 3T Philips Achieva MRI Scanner (Cincinnati Children’s Hospital Medical Center)

##### 151 participants

High-resolution T1-weighted anatomical images were acquired (TR: 8.1s, TE: 2.17ms, FOV: 25.0 cm × 21.1 cm × 18.0 cm, matrix: 252 × 211, flip angle: 8 degrees, slice thickness: 1 mm). Functional T2^∗^-weighted images were obtained using the HUSH silent clustered-sparse temporal image acquisition (TR: 2000 ms, TE: 38 ms, FOV: 24.0 cm × 24.0 cm × 12.8 cm, flip angle: 90 degrees, matrix: 64 × 64, slice thickness: 4 mm, 32 axial slices with 0% distance factor; voxel size: 3.75 mm × 3.75 mm × 4 mm).

#### 3T Siemens Magnetom Allegra MRI Scanner (University of Alabama at Birmingham)

##### 23 participants

High-resolution T1-weighted anatomical images were acquired (TR: 2.3 s, TE: 2.17 ms, FOV: 25.6 cm × 25.6 cm × 19.2 cm, matrix: 256 × 256, flip angle: 9 degrees, slice thickness: 1 mm). Functional T2^∗^-weighted images were obtained with the HUSH silent clustered-sparse temporal image acquisition (TR: 2000 ms, TE: 35 ms, FOV: 24.0 cm × 24.0 cm × 12.8 cm, flip angle: 70 degrees, matrix: 64 × 64, slice thickness: 4 mm, 30 axial slices with 7% distance factor; voxel size: 3.8 mm × 3.8 mm × 4 mm).

fMRI stimuli were presented using an event-related design as described in detail previously (Allendorfer et al., 2012a; Vannest et al., 2012, 2015). Briefly, for each stimulus, a word pair was presented for 6 s and participants were instructed to read the second word out loud; audio responses were recorded. This was followed by 6 s of data collection (3 image volumes) with the word “STOP,” instructing participants to stop talking during acquisition. A total of 180 whole-brain volumes were collected across the full 12 min of the task. The task was not dependent on a verbal response: if there was no response after a word pair was presented, the task would continue uninterrupted (Vannest et al., 2012, 2015).

### fMRI Data Preprocessing

**FIGURE 3:**
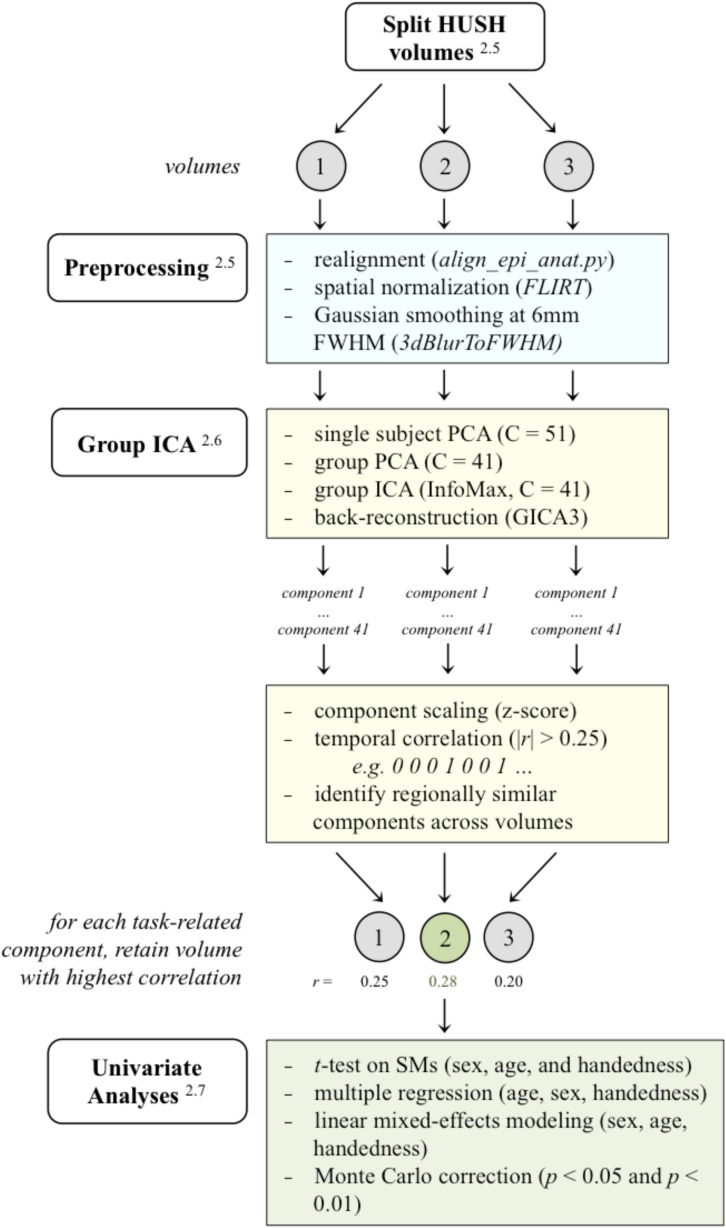
A schematic of the analysis pipeline. HUSH volumes were split into the 3 volumes acquired, and subsequent preprocessing and analysis in GIFT (PCA/ICA) was applied to volumes separately. After sorting components using the binary task time series, regionally similar components were identified across the three analyses and the volume with the highest correlated to the task time series was retained for further analyses. (Superscripts correspond to sections in the methods that further describe that stage in the analysis. HUSH, Hemodynamics Unrelated to Sounds of Hardware; FLIRT, FMRIB’s linear image registration tool; FWHM, full width at half maximum; PCA, principle component analysis; ICA, independent component analysis; SMs, spatial maps).

Data were processed using Analysis of Functional NeuroImages software (AFNI; Cox, 1996) and FMRI software library (FSL; Smith et al., 2004). Functional images were first split into three separate parts: the first, second, and third volumes for each stimulus presentation were separated into three separate functional images in order to account for signal intensity changes in the hemodynamic response function over time (Schmithorst and Holland, 2004). See Figure 3 for a schematic of the analysis pipeline. Functional images were then motion corrected using AFNI’s *align_epi_anat.py* and *3dvolreg*. Functional images were registered to the anatomical images using FSL’s FLIRT (Jenkinson et al., 2002), resampled to 3 mm isotropic voxels, and standardized to the MNI152 template atlas using FSL’s non-linear registration tool (FNIRT). We smoothed all participants’ datasets to an effective smoothness of a Gaussian FWHM of 6 mm using AFNI’s *3dBlurToFWHM*. Functional volumes did not undergo any additional filtering or artifact regression prior to Group ICA (Calhoun et al., 2001). Trials were not excluded based on participant responses. Since participants undergo the process of encoding word paired associates whether or not they produce the correct word in the scanner, all trials were used for each subject in the second level analyses.

### Group ICA

Group spatial ICA was carried out using GIFT in Matlab software (Group ICA fMRI Toolbox, v4.0b) for each of the three image volume sets by first conducting two principle component analyses (PCAs) for data reduction. Subject-specific PCA was conducted as the first round of PCA to reduce each subject’s functional data, yielding 51 components. Subjects’ data were then temporally concatenated and underwent a second round of PCA, yielding 41 components (Calhoun et al., 2001; Erhardt et al., 2011). Group level independent components were derived using the Infomax ICA algorithm, yielding 41 group level components (for each of the three HUSH image volumes).

In order to maximize discrimination of different signal sources, segment task-related brain activity into functionally relevant and interpretable sources, and improve the detection of noise sources and minimize the effect of motion artifacts in task-related components, a high model order of 41 independent components was employed (Ystad et al., 2010; Ray et al., 2013; Saliasi et al., 2014; Hutchison and Morton, 2015). The number of independent components used in the present study consistent with a similar previous study from our group (Vannest et al., 2015).

Subject-specific spatial maps (SMs) were derived with GIFT’s GICA3 back-reconstruction method (Calhoun et al., 2001, 2002; Erhardt et al., 2011). GICA3 estimates subject-specific time-courses and SMs from mixing matrices derived in PCA data reduction steps, and has been shown to provide more robust results with more intuitive interpretation (Erhardt et al., 2011).

GIFT’s temporal sorting tool allows for the classification of components by temporal characteristics, comparing the model’s time course to the time courses of independent components (Rachakonda et al., 2007). Using the correlation function in the temporal sorting tool, group-level components were sorted by binary task time series (model time course) and components with a correlation coefficient |*r*| > 0.25 were identified as task-related components. The binary task time series designated “1” for the “generate” condition (active generation, task-positive) and “0” for the “read” condition (passive reading, task-negative). See Figure 3 for an example of the binary task time series used.

After temporal sorting of components, visual examination of components with the highest positive and negative correlations suggested that outside of |*r*| > 0.25, some components appeared to capture noise (cerebrospinal fluid in ventricles, as well as ringing and lower overall intensity with scattered regional contributions). To capture true regional, task-related activity, the correlation threshold for the present study was set at |*r*| > 0.25. For the “generate” condition, component correlations were between 0.357 > *r* > 0.424, and for the “read” condition, correlations were between −0.260 > *r* > −0.302.

Components with a correlation coefficient of *r* > 0.25 were identified as task-positive (correlating with the “generate” condition), and components with a correlation coefficient of *r* < −0.25 were identified as task-negative (correlating with the “read” condition) and were retained. Components with a correlation coefficient between −0.25 > *r* > 0.25 were excluded from all further analyses. Components were visually inspected and regionally similar/matching components were identified across all three image volumes. If a component met threshold for task-relatedness (|*r*| > 0.25) across more than one of the three image volumes, then the most highly correlated volume was selected for further analysis. These six derived task-related components represent statistically independent sources contributing to task-positive and task-negative networks and do not reflect a specific contrast within the task (generate vs. read).

### Relationships Between Task-Related Components and Sex, Handedness, and Age

To compare differences in network extent among our participants, we utilized an approach available within the group ICA toolbox (GIFT). GIFT produces subject specific SMs for each independent component by implementing a series of back-reconstruction steps from each component at the group level (Calhoun et al., 2001; Meier et al., 2012). To investigate the effects that sex, handedness, and age may have on task-positive and task-negative networks, we conducted a second level analysis by extracting SMs from each subjects’ individual dataset for each corresponding task-related component (components that met a correlation threshold of |*r|* > 0.25).

A total of six components met threshold for task-relatedness, and participants corresponding SMs were used in a series of two-sample *t*-tests using AFNI’s *3dttest*++. We used two-sample *t*-tests to examine any spatial differences in network extent of task-related components between sexes (male vs. female), handedness (atypical vs. right), and age (<50 vs. ≥50 years old) groups, (including scanner type as a covariate). We also explored whether any differences between sexes could be attributed to handedness; specifically, we ran separate two-sample *t*-tests comparing males and females (one for right-handed individuals, one comparing atypically handed individuals).

To examine age as a continuous variable, as well as the effect of sex differences while controlling for scanner and handedness, we conducted multiple regression analyses using AFNI’s *3dRegAna*. All analyses were corrected for multiple comparisons using Monte Carlo simulations with results considered significant if clusters met the threshold of *p* < 0.05 when corrected for multiple comparisons (AFNI’s *3dClustSim* yielded: cluster size at least 171 voxels when corrected at *p* < 0.05 and 80 voxels when corrected at *p* < 0.01). Additionally, we investigated if sex differences change with age across all task-related components using a linear mixed-effects modeling approach with AFNI’s *3dLME*, with our model specification testing for an interaction effect between sex and age, while controlling for scanner and handedness.

## Results

### Performance Data

A Wilcoxon signed-rank test conducted in SPSS Statistics 25 showed that post-test accuracy for the “generated” words [M(SD),%: 22.57(3.7), 75.2%] and the “read” words [21.75(3.9), 72.5%] was significantly different after encoding (*Z* = −2.643, *p* = 0.008). Independent-samples *t*-tests revealed no significant differences between sexes or handedness groups for memory of read words (*p* = 0.099 and *p* = 0.863, respectively) or generated words (*p* = 0.135 and *p* = 0.219, respectively). For memory of “read” words, differences were found between older [M(SD),%: 20.67(4.0), 68.9%] and younger [22.23(3.8), 74.1] adults (*p* = 0.014), as well as memory of “generated” words between older [21.46(3.9), 70.2%] and younger [23.07(3.5), 75.5%] adults (*p* = 0.008). Multiple linear regressions revealed no sex differences in post-test accuracy after controlling for age. Sex was not a predictor of memory performance on generated words while controlling for age and handedness *F*(3,169) = 1.387, *p* = 0.303, *R*^2^ = 0.024. Sex was also not a significant predictor of memory performance on read words while controlling for age and handedness *F*(3,169) = 1.758, *p* = 0.058, *R*^2^ = 0.030. Additionally, there were no interaction effects of age and sex on performance of generated words *F*(4,168) = 1.109, *p* = 0.590, *R*^2^ = 0.026, or on read words *F*(4,168) = 1.377, *p* = 0.613, *R*^2^ = 0.032.

### Group ICA: Task-Positive Components

**FIGURE 4:**
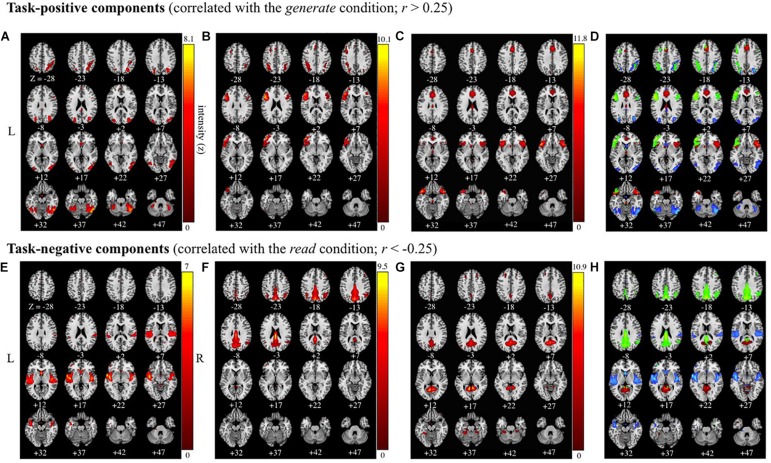
Task-positive and task-negative components (correlated with the *generate* and *read* conditions |*r*| > 0.25). Images are presented in neurological orientation (R = R). Group average independent components positively correlated (*r* > 0.25), **(A–C)**, and negatively correlated (*r* < –0.25), **(E–G)** with the task time course, along with composite images for task-positive **(D)** and task-negative **(H)** components presented below. Components span the following brain regions: **(A)** bilateral occipital; **(B)** bilateral (left-lateralized) inferior frontal gyrus and precentral gyrus; **(C)** anterior insula and superior temporal gyrus; **(D)** composite image of task-positive components **(A–C)**; **(E)** bilateral superior temporal gyrus; **(F)** bilateral precuneus and posterior cingulate cortex; **(G)** posterior cingulate cortex and culmen; **(H)** composite image of task-negative components **(E–G)**.

Of the 41 components, three components were identified as task-positive, meeting a threshold of *r* > 0.25 for the generate condition (Figures 4A–C and Table 2). The component with the highest correlation with the task (*r* = 0.4244) included bilateral fusiform gyri, bilateral declive, right inferior temporal gyrus, middle occipital gyrus, middle temporal gyrus (MTG), precuneus, and superior parietal lobule. The second highest correlated task-positive component (*r* = 0.3784) included left middle frontal gyrus, bilateral IFG, left ventral anterior insula, left precentral gyrus, and left inferior parietal lobule (IPL). The third component (*r* = 0.3573) included bilateral IFG, bilateral superior temporal gyri (STG), cingulate gyrus, anterior cingulate cortex, and bilateral ventral anterior insula.

**TABLE 2:** MNI coordinates in task-positive and task-negative components.

**Component**	**r**	**Hush volume**	**Location**	***X***	***Y***	***Z***

**Task-positive components** (Figure 4)						
Figure 4A	0.4244	2	L fusiform gyrus	–46	–58	–16
			R fusiform gyrus	40	–66	–20
			R interior temporal gyrus	50	–56	–16
			L declive	–32	–58	–20
			R declive	38	–58	–22
			L middle occipital gyrus	–32	–84	6
			R middle occipital gyrus	42	–71	–16
			R MTG	54	–58	–14
			L precuneus	–24	–70	36
			R precuneus	32	–72	34
			L superior parietal lobule	–22	–66	48
			R superior parietal lobule	24	–64	44
			R IPL	36	–54	50
			L cuneus	–26	–78	28
			L culmen	–30	–50	–22
			R culmen	36	–52	–24
Figure 4B	0.3784	2	L middle frontal gyrus	–44	40	–2
			L IFG	–46	26	18
			R IFG	46	30	14
			L ventral anterior insula	–46	10	12
			L precentral gyrus	–50	12	8
			L IPL	–34	–56	42
			R IPL	36	–58	46
			L STG	–46	16	–8
			L medial frontal gyrus	–2	28	38
			L cingulate gyrus	–2	24	40
Figure 4C	0.3573	2	L IFG	–40	18	–12
			R IFG	44	16	–8
			L STG	–44	16	–12
			R STG	44	16	–12
			L cingulate gyrus	0	22	36
			R cingulate gyrus	2	24	32
			L ACC	0	32	22
			R ACC	2	36	22
			L insula	–40	14	–4
			R insula	36	18	0
**Task-negative components** (Figure 4)						
Figure 4E	–0.3019	2	L posterior insula	–44	–4	–6
			R posterior insula	44	–12	4
			L STG	–56	4	–4
			R STG	48	–2	–4
			L transverse temporal gyrus	–40	–24	10
			R transverse temporal gyrus	48	–24	10
			R precentral gyrus	48	–14	6
			R postcentral gyrus	56	–26	14
Figure 4F	–0.2740	2	L precuneus	0	–72	36
			R precuneus	2	–72	40
			L cuneus	0	–72	32
			R cuneus	4	–72	32
			L cingulate gyrus	0	–26	28
			R cingulate gyrus	2	–44	32
			L PCC	–4	–38	22
			R PCC	4	–36	22
			L IPL	–34	–58	40
			R IPL	40	–58	44
			L angular gyrus	–34	–58	36
			R angular gyrus	44	–60	34
			L supramarginal gyrus	–44	–56	26
Figure 4G	–0.2607	3	L PCC	–8	–56	4
			R PCC	10	–54	4
			L culmen	–6	–46	0
			R culmen	6	–46	–2
			L parahippocampal gyrus	–10	–50	0
			L lingual gyrus	–12	–54	2
			L precuneus	–4	–62	16
			R precuneus	4	–64	20
			L fusiform gyrus	–26	–40	–16
			L superior frontal gyrus	–18	34	36
			L middle frontal gyrus	–22	22	42
			R middle frontal gyrus	26	20	44

*L, left; R, right; MTG, middle temporal gyrus; IFG, inferior frontal gyrus; IPL, inferior parietal lobe; STG, superior temporal gyrus; ACC, anterior cingulate cortex; PCC, posterior cingulate cortex.*

### Group ICA: Task-Negative Components

Three components were identified as task-negative, meeting a threshold of *r* < −0.25 for the read condition (Figures 4D–F and Table 2). Components are listed from highest correlation: the first component (*r* = −0.3019) included bilateral posterior insula, STG, transverse temporal gyri, and right pre- and post-central gyri. The second component (*r* = −0.2740) included bilateral cuneus and precuneus, cingulate gyri, posterior cingulate cortex, and right IPL. The third component (*r* = −0.2607) included posterior cingulate cortex and left culmen.

### Relationships Between Task-Related Components, Sex, Handedness, and Age

**FIGURE 5:**
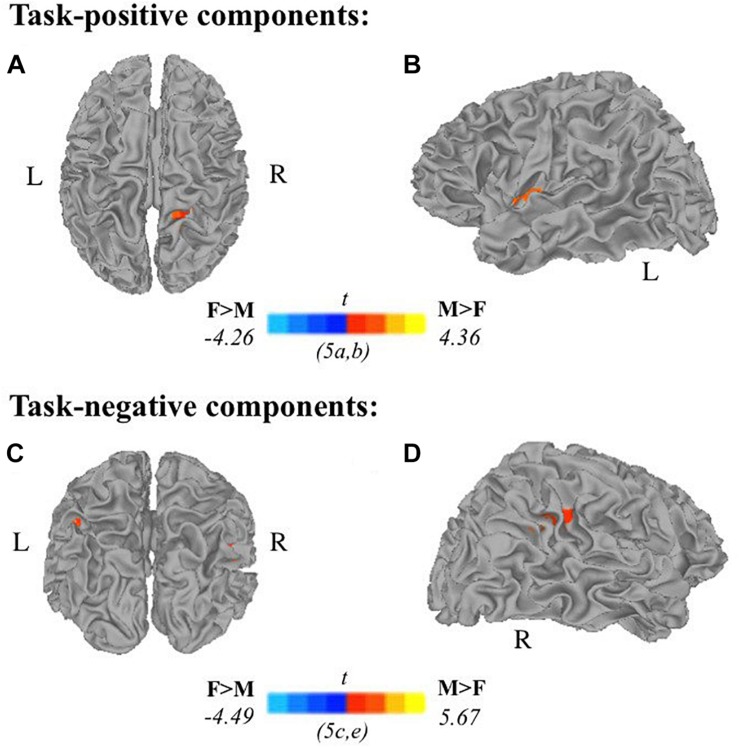
Relationships between sex and task-related components: Two-sample *t*-tests between males and females in task-positive components **(A,B)** and task-negative components **(C,D)** reveal males recruit additional brain areas during active encoding compared to females: **(A)** right postcentral gyrus, **(B)** left insula; and during passive encoding: **(C)** left supramarginal gyrus, **(D)** right superior temporal gyrus (F, females; M, males; *p* < 0.05 corrected).

**FIGURE 6:**
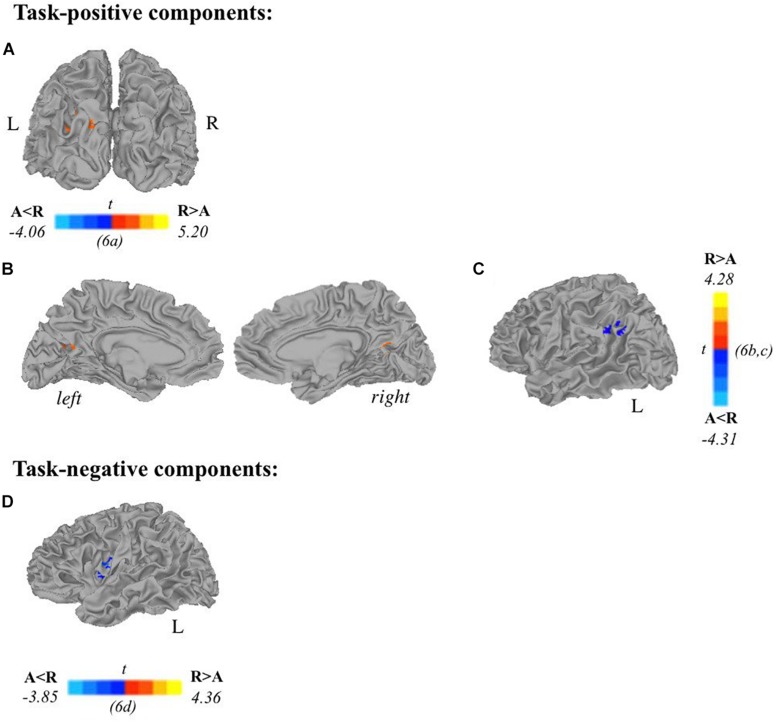
Relationships between handedness and task-related components. Two-sample *t*-tests reveal differences between atypically handed and right-handed individuals in task-positive components **(A–C)** and task-negative components **(D)**. In terms of spatial extent of task-related activation, atypical-handers and right-handed individuals recruited additional regions during active encoding: **(A)** left cuneus, **(B)** posterior cingulate cortex, **(C)** left angular gyrus; and during passive encoding: **(D)** left insula. Orange depicts areas where atypical-handers task-related extent was greater than right-handed individuals, and blue depicts areas where right-handed individuals task-related extent was greater than atypical-handers (A, atypical-handed; R, right-handed; *p* < 0.05 corrected).

Two-sample *t*-tests revealed differences in spatial extent in task-related brain activity recruited between sexes during self-generation and passive reading. All results presented meet a threshold of *p* < 0.05 corrected. For males compared to females during self-generation, network extent was greater in right postcentral gyrus (Figure 5A) and left dorsal anterior insula (Figure 5B), and in left supramarginal gyrus (Figure 5C) and right STG (Figure 5D) during reading. For right-handers compared to atypical-handers, extent was larger in left insula (Figure 6D) and angular gyrus (Figure 6C) during reading and self-generation, respectively, and smaller in left cuneus (Figure 6A) and posterior cingulate cortex (PCC; Figure 6B) during generation.

Separate analyses of right- and atypically handed individuals’ between-group sex differences showed somewhat different effects across groups. Right-handed males showed increased recruitment in right middle occipital gyrus compared to right-handed females during self-generation, while atypically handed individuals did not show this sex effect. Right-handed females also showed increased recruitment in right superior temporal gyrus compared to males during self-generation that was not seen among atypically handed individuals. During reading, right-handed females showed increased recruitment of right middle frontal gyrus and right cuneus compared to right-handed males, but this difference was not seen among atypically handed individuals.

**FIGURE 7:**
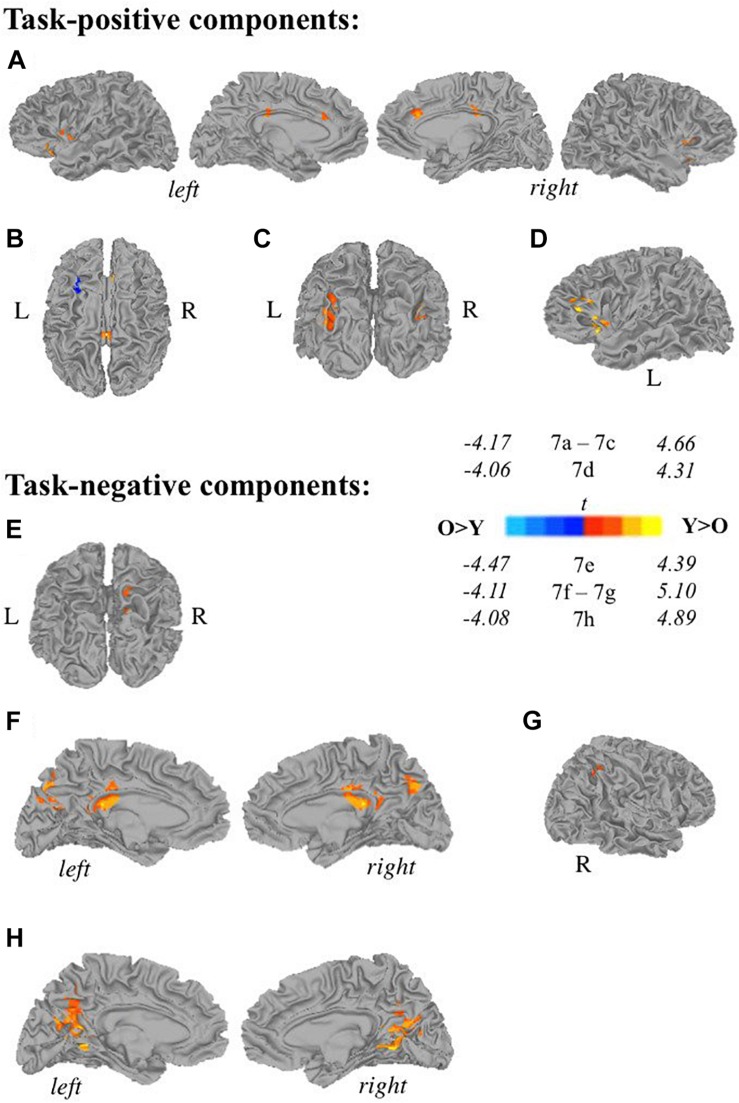
Relationships between age and task-related components. Two-sample *t*-tests reveal differences between younger (<50 years old) and older (≥50 years old) adults in task-positive components **(A–D)** and task-negative components **(E,F)**. Recruitment among young adults seemed more widespread compared to older adults during self-generation, with greater recruitment across **(A)** bilateral insula, anterior and middle cingulate cortices, and left inferior frontal gyrus, **(C)** bilateral middle occipital gyrus, **(D)** left inferior frontal gyrus and left insula. During self-generation, older adults showed greater recruitment compared to younger adults in panel **(B)** left middle frontal gyrus. During reading, younger adults showed greater activity compared to older adults across: **(E)** right precuneus, **(F)** bilateral precuneus and PCC, **(G)** right inferior parietal lobe, and **(H)** bilateral PCC [Y, younger adults (<50 years old), O, older adults (≥50 years old); *p* < 0.05 corrected].

Younger adults (<50 years old) displayed more widespread involvement during both self-generation and passive reading compared to older adults (≥50 years old) across a range of areas (Figures 7A,C–H) except for left middle frontal gyrus (Figure 7B), which showed greater recruitment during self-generation among older compared to younger adults (corrected *p* < 0.05). During self-generation, younger adults showed greater recruitment across bilateral insula, anterior and middle cingulate cortices (Figure 7A), and left IFG (Figures 7A,D), and bilateral middle occipital gyri (Figure 7C). During passive reading, younger adults showed greater involvement across right precuneus (Figure 7E), bilateral precuneus (Figure 7F), posterior cingulate cortices (Figures 7F,H), and right inferior parietal lobe (Figure 7G).

### Regression and Mixed-Effects Modeling Results

**FIGURE 8:**
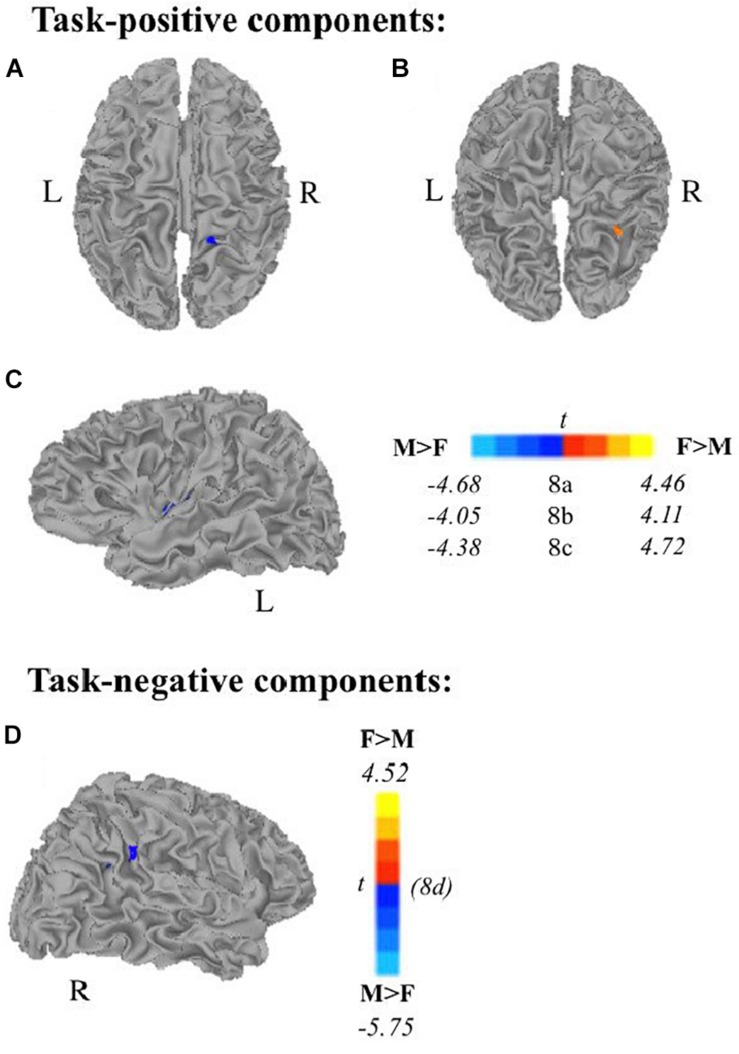
Relationship between sex and task-related components, controlling for scanner, age, and handedness. Males show greater task-positive network extent when controlling for scanner, age, and handedness in panel **(A)** right paracentral lobe and **(C)** left insula during self-generation and greater task-negative network extent in panel **(D)** right supramarginal gyrus/STG during passive reading. Females show greater task-positive recruitment during self-generation of panel **(B)** right middle temporal gyrus (F, females; M, males; *p* < 0.01 corrected).

Multiple linear regression analyses allowed us to examine (1) age as a continuous variable while controlling for scanner type, and (2) the effect of sex on task-related components while controlling for age, handedness, and scanner type. All results presented meet a threshold of *p* < 0.01 corrected. Subjects’ extracted z-scores represent deviation from the group level within that component. Self-generation areas showed decreased recruitment (via subject specific extracted z-scores) with increasing age across frontal and temporo-parietal areas. Passive encoding areas including STG, PCC, and IPL also showed decreased recruitment as age increases. We also found that during self-generation, males recruited right paracentral areas (Figure 8A) and left dorsal anterior insula (Figure 8C) more than females did, while females recruited right middle temporal gyrus (Figure 8B) more than males did. During passive reading, controlling for age, handedness, and scanner, men recruited right supramarginal gyrus and superior temporal gyrus areas (Figure 8D) more than women did.

**FIGURE 9:**
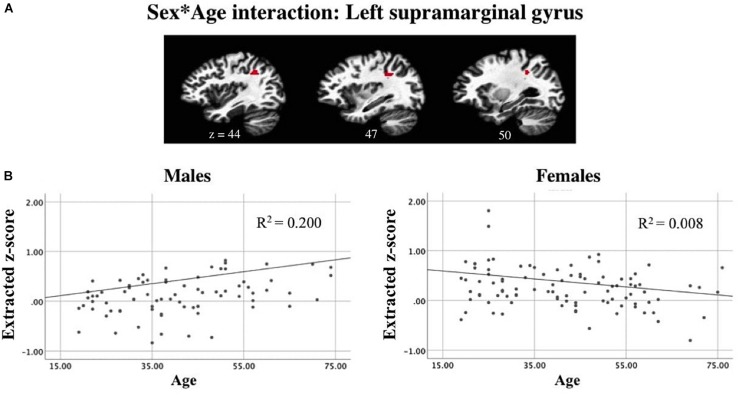
Linear mixed-effects model revealed **(A)** an interaction between sex and age while controlling for scanner and handedness in the left supramarginal gyrus in a task-positive component. Correlations between subjects’ mean z-score for the left supramarginal gyrus ROI and age suggests that **(B)** as age increases, males show increased recruitment of left SMG during passive reading while females show a decline in BOLD signal intensity as age increases (*p* < 0.01 corrected).

A linear mixed-effects model examining task-related components revealed a significant interaction between sex and age while controlling for scanner and handedness in task-positive networks (Figure 9A). In the active generation condition, as age increases, males showed increased recruitment of the left supramarginal gyrus. In contrast, for females, the slope of the trend line shows an opposite pattern: decreased recruitment as age increases. Presented results meet a corrected threshold of *p* < 0.01. The relationship between individual subjects’ age and average z-score extracted from the left SMG are shown for males and females separately, with the regression line displayed controlling for handedness and scanner to show direction of the effect (Figure 9B).

## Discussion

We examined network differences underlying active and passive memory encoding using a verbal paired-associate learning task. Overall, the results of the analyses were consistent with previous behavioral studies and indicate that generated words during the encoding task were remembered more accurately in post-testing than read words were (Schefft and Biederman, 1990; Olofsson and Nilsson, 1992; Vannest et al., 2012, 2015). The inclusion of both semantically and phonologically related words might have interacted with the recognition of read and generated words to some degree (Siegel et al., 2012), but this effect is likely small and, thus, not investigated here. ICA revealed a broad fronto-parietal network underlying self-generation, while passive reading showed strong temporal-occipital contributions. Further investigation of self-generation and passive reading showed sex, age, and handedness differences in regional patterns of network involvement.

### Sex, Age, and Handedness Differences in Active vs. Passive Learning

#### Sex Effects

There is a large body of work suggesting that networks supporting semantic and phonological processing may be more left lateralized in healthy, right-handed males compared to females who tend to show more bilateral and widespread pattern of network involvement (Binder et al., 2009). In previous studies, this differential sex effect was seen in superior and middle temporal areas during a story-listening task (Kansaku et al., 2000). However, we did not observe this effect during passive reading, where a greater spatial extent of task-negative related activation in the right superior temporal gyrus was observed in males compared to females. Considering the role personal handedness plays in the neural organization of language (Kansaku and Kitazawa, 2001; Szaflarski et al., 2002, 2006; Tzourio-Mazoyer et al., 2010), and the inclusion of only (or predominantly) right-handed participants in studies of sex differences in language distribution, our findings in a large sample of right- and atypical-handers may reflect differential recruitment during semantic or phonological processing than previously observed (Andreano and Cahill, 2009). Further investigation of sex differences between right- and atypically handed individuals revealed that during self-generation, right-handed females show increased recruitment in right superior temporal gyrus compared to right-handed males that was not seen in the atypically handed group. Compared to right-handed males, right-handed females also showed right-lateralized activity in the middle frontal gyrus during reading, which was not seen among atypically handed individuals. While females have demonstrated more bilateral involvement in posterior temporal areas during linguistic processing compared to males, females have shown a similar left-lateralized pattern to men in the angular and supramarginal gyri (Kansaku et al., 2000). Our findings reveal greater recruitment among males in left supramarginal gyrus while reading compared to females. During generation, recruitment of the left SMG interacted with sex and age such that males showed greater left SMG recruitment as age increased while females did not. These patterns reflect differential recruitment of resources based on sex and age during both passive and active encoding (Stoeckel et al., 2009; Oberhuber et al., 2016).

#### Age Effects

During self-generation, older adults showed reduced recruitment of frontal areas, including left inferior frontal gyrus and bilateral insula, as well as bilateral middle occipital gyrus, compared to younger adults. These findings of left IFG engagement during active encoding among younger but not older adults are consistent with previous reports of greater prefrontal lateralization among younger adults (Cabeza, 2002; Morcom et al., 2003). This age-related reduced lateralization of task-related activity may reflect a decreased specialization of brain areas relevant for task demands (Cabeza, 2002). One region implicated among older compared to younger adults was the left middle frontal gyrus. Increased recruitment of left MFG among older adults may represent a compensatory mechanism (Morcom et al., 2003) during semantic processing necessary for similar performance.

During passive reading, older adults exhibited reduced recruitment compared to younger adults across posterior and parietal brain regions including bilateral precuneus, posterior cingulate cortex, and right inferior parietal lobe. This is in contrast to previous studies indicating older adults involve posterior brain regions to a greater extent compared to younger adults (Morcom et al., 2003). Our findings of greater PCC recruitment in younger compared to older adults suggests passive reading aloud may be more effortful for older adults, or even simply that passive reading engages different networks among younger vs. older adults, reflecting differential strategies between the groups (Berlingeri et al., 2013). This compensation view is contrasted with a dedifferentiation perspective of the age-related differences in brain activity. The CRUNCH (Compensation-Related Utilization of Neural Circuits Hypothesis) model provides a framework for age-related increases in activation in different brain areas, not specific to hemisphere (Reuter-Lorenz and Cappell, 2008; Berlingeri et al., 2013). The level of task demand across studies also likely plays a significant role in the CRUNCH model (Berlingeri et al., 2013). When engaging in tasks with overall lower cognitive load, neural effects supporting the CRUNCH model may not be elucidated (Jamadar, 2018).

An examination of age as a continuous variable yielded several components showing age-related decreases across both self-generation and passive reading. Overall, two out of three task-positive components showed age-related decreases (Figures 4A,C) indicating that when age increases, our relatedness measure decreases across inferior frontal, superior temporal, and superior parietal brain areas. Other studies have supported age-related decreases in parietal areas across various cognitive tasks (Grady et al., 2010), and during self-generation (Vannest et al., 2015). Two out of three task-negative components also showed age-related decreases (Figures 4F,G) across temporal and posterior cingulate areas during reading, consistent with evidence of decreased default mode network activity among older individuals (Grady et al., 2010; Mevel et al., 2013; Vannest et al., 2015).

#### Handedness Effects

Handedness plays an important role in hemispheric language dominance (Geschwind and Galaburda, 1985; Szaflarski et al., 2002, 2012). Neuroanatomical differences exist between right-handed and atypically handed individuals, particularly in the planum temporale (Foundas et al., 1995, 2003; Shapleske et al., 1999). Studies also show a negative relationship between corpus callosum volume and degree of handedness, where increasing atypical handedness is associated with larger corpus callosum volumes (Habib et al., 1991; Witelson and Goldsmith, 1991). In turn, this may be influenced by increased interhemispheric information transfer among atypical- compared to right-handers (Tzourio-Mazoyer et al., 2010; Gao et al., 2015; Sahu et al., 2016). Interhemispheric communication time has been linked to brain volume, and evidence suggests those with larger brains have been shown to group quick cognitive functions in one hemisphere (Ringo et al., 1994). Our findings of right-handers compared with atypical-handers showing left angular gyrus involvement during self-generation and left insular involvement during passive reading may be due to the left-dominant language processing seen among right-handers, while atypical-handers may have substantial interhemispheric communication to lighten processing load.

While left angular gyrus activation has been associated with language ability (Van Ettinger-Veenstra et al., 2016) and semantic processing (Seghier and Price, 2013; Hartwigsen et al., 2016), examination of functional properties and subdivisions of the angular gyrus reveals an integrative role across multisensory domains, including reorienting attention and familiar problem solving (Seghier and Price, 2013). Activation of left angular gyrus may also be modulated by several factors, including reading level and age (Meyler et al., 2007, 2008), but sex and handedness have not been studied in depth as potential modulatory factors of the angular gyrus (Seghier and Price, 2013). Recruitment of the left angular gyrus among right-handers during self-generation may be related to increased reliance on left dominant semantic processing areas compared to atypical handers. Gray matter asymmetry between left- and right- handed individuals suggests less specialization for speech in the left hemisphere among left-handed individuals (Hervé et al., 2006), and right-handed individuals have shown more left lateralized patterns of activity compared to left-handed individuals (Gao et al., 2015), suggesting organizational differences in semantic processing between left- and atypically handed individuals.

#### Sex Effects, Controlling for Age and Handedness

Our analyses of sex effects on task-related components while controlling for age, handedness, and scanner revealed a similar pattern of involvement of the left dorsal anterior insula during self-generation among males and right middle temporal gyrus among females. We also found right hemisphere differences among sexes (males > females) in the paracentral lobe during self-generation and in the supramarginal gyrus during passive reading. The role of the anterior insula in affective and cognitive functions suggests that our findings of increased involvement of left dorsal anterior insula among males compared to females may be related to a lateralization effect of performance monitoring (Dosenbach et al., 2006), attention orienting (Corbetta and Shulman, 2002), or salience (Seeley et al., 2007; Menon and Uddin, 2010). Our previous study examining verb generation suggested sex lateralization effects may be dependent on performance and language ability, and found a similar right lateralized pattern for males in caudate/anterior cingulate gyrus when controlling for performance (Allendorfer et al., 2012a). Another study investigated the potential relationship between white matter integrity of the superior longitudinal fasciculus (SLF) and language functioning across healthy, right-handed participants ranging from 19–76 years old (Madhavan et al., 2014). This study found a differential pattern of decline in fractional anisotropy (FA) of the SLF in relation to age in males and females, as well as with language functioning as measured by performance on the Controlled Oral Word Association Test.

### Attention and Salience During Encoding Implicates a Fronto-Parietal Network

A “network” approach to functional organization in the brain suggests the brain responds to environmental demands (sensory or cognitive) by recruiting brain areas that aid in signal processing. This large-scale distribution of resources results in sets of regions showing statistical dependence in relation to the specific demand or task. More recent approaches to cognitive neuroscience involve a framework with several differentiated and interacting networks underlying human brain function (Menon and Uddin, 2010) with these networks having specific profiles of activation and deactivation.

A fronto-parietal network, including the posterior parietal cortex (PPC) and areas of the prefrontal cortex (Buckner et al., 1999; Otten et al., 2001), has been shown to underlie visual attention (Corbetta et al., 2002, 2008). Involvement of the inferior parietal lobe (IPL) in this network may be related to maintaining attention on task goals and encoding events in the environment (Rueckert and Grafman, 1998; Adler et al., 2001; Vandenberghe et al., 2001; Singh-Curry and Husain, 2009) or performance (Donnelly et al., 2011). In this study, task-positive components underlying self-generation showed broad fronto-parietal involvement, including left IPL (Figure 4B), suggesting an increase in attentional demands when self-generating compared to reading. One study examined how differential attention during verbal encoding modulates fronto-parietal brain activity and found significant contributions from the middle frontal gyri (MFG) during high-attention stimuli (Christensen et al., 2012). The MFG has been hypothesized to be an area of integration between dorsal and ventral attention streams, serving as a gateway between top–down and bottom–up attention and playing a major role in controlling and reorienting attention (Japee et al., 2015). In this study, generating the second word in the pair involves using the presented cue and a top–down search of known words semantically or phonologically related to the first word. Our findings of left MFG recruitment during self-generation suggests increased allocation of resources to this brain region during active encoding.

The “salience network” refers to a group of brain regions involved in cognitive or emotional arousal; it includes the anterior insula, dorsal anterior cingulate cortex (dACC), and several subcortical and limbic structures (Seeley et al., 2007; Morgan et al., 2008; Kay et al., 2012; Menon, 2015). Implication of the insular cortex in neuroimaging studies suggests its role in salience and stimuli detection, facilitating attention and working memory during task switching (Sridharan et al., 2008; Menon and Uddin, 2010). In this study, the insula and anterior cingulate cortex were identified in task-positive components, likely contributing to sustained attention during self-generating word pairs. Our examination of sex differences suggests males may recruit brain areas involved with sustained attention and task switching attention more than females do during self-generation. The two clusters in left insula and right postcentral gyrus that were involved with self-generation among males compared to females may be serving as an attention modulating mechanism when task demands increase during active encoding (Steinmetz et al., 2000). Similar sex-differences were observed in developmental but not adult studies of language lateralization (Szaflarski et al., 2002, 2012). The insula was also implicated in our examination of handedness and brain regions underlying reading aloud in that right-handers showed greater extent in the left insula involvement compared to atypical-handers. A study of effective connectivity during a Chinese semantic task found that left-handers showed differential effective connectivity between the insula and prefrontal/occipital areas compared to right-handers, suggesting differential information processing among atypical-handed individuals during visual and semantic word retrieval of Chinese characters (Gao et al., 2015). Due to the integrative role of the insula in bottom–up and top–down processing, these authors hypothesized that handedness may impact information transfer at a causal, system level during semantic word retrieval.

### A Dynamic Network Underlies the Encoding Process

A meta-analysis of 74 fMRI encoding and memory studies revealed five main brain areas consistently associated with subsequent memory. The study also examines how patterns of activity are modulated by task-related conditions (nature of material: verbal or pictorial; type of encoding: item or associative) (Kim, 2011). The findings of this study supported the “task-dependency” principle, specifically, that the encoding process cannot be reduced to a fixed set of brain areas, but rather the neural correlates of encoding should be viewed as a dynamic network that responds to task-specific requirements (Otten and Rugg, 2001; Rugg et al., 2002; Kim, 2011). Therefore, it is the type of encoding task that determines which regions are functionally relevant and will be recruited for successful processing and will show subsequent memory effects.

Certain aspects of network organization and recruitment during encoding may be dependent on specific demographic variables such as age (Szaflarski et al., 2006, 2012; Allendorfer et al., 2012b; Maillet and Rajah, 2014; Chastelaine et al., 2017), sex (Mulligan and Lozito, 2004; Hill et al., 2014), or handedness (Seghier and Price, 2013). Our findings are consistent with differential recruitment of these dynamic networks during self-generation based on age, handedness, and sex. In task-positive networks, older adults showed reduced prefrontal lateralization compared to younger adults as left IFG showed greater spatial extent of activation among younger adults than older adults. Right-handed individuals also displayed greater recruitment of left semantic processing areas compared to left-handed individuals in these same task-positive networks. The influence of sex on task-related networks suggests increased right hemisphere recruitment among males compared to females during both self-generation and passive reading. These findings provide some insight into networks underlying active and passive encoding, and how the recruitment of these dynamic networks may be influenced by demographic factors like age, sex, and preferred handedness.

## Conclusion

In summary, ICA of fMRI data collected during a verbal paired-associate learning task revealed fronto-parietal network contributions during self-generation of word pairs, and recruitment of temporo-occipital areas during reading words aloud. Sex, handedness, and age groups showed similar memory performance, but significant differences in task-positive and task-negative brain areas across groups suggest differential recruitment of encoding network areas to achieve similar performance levels.

## Ethics Statement

The Institutional Review Boards at the University of Cincinnati, the Cincinnati Children’s Hospital Medical Center, and the University of Alabama at Birmingham approved this project (NIH R01-NS04828), and all participants provided written informed consent. No vulnerable populations were included in this study.

## Author Contributions

JS collected the funds. SN performed the literature search. JV and JS designed the study. SN, RN, AG, and DMperformed statistical analysis. SN, RN, JA, AG, and JS interpreted the data. SN, and JS prepared the manuscript. RN, JA, AG, JV, and DM reviewed the manuscript and approved the content.

## Conflict of Interest Statement

The authors declare that the research was conducted in the absence of any commercial or financial relationships that could be construed as a potential conflict of interest.
